# Study protocol: evaluation of sheds for life (SFL): a community-based men’s health initiative designed “for shedders by shedders” in Irish Men’s sheds using a hybrid effectiveness-implementation design

**DOI:** 10.1186/s12889-021-10823-8

**Published:** 2021-04-26

**Authors:** Aisling McGrath, Niamh Murphy, Noel Richardson

**Affiliations:** 1 Department of Sport and Exercise Science, Waterford Institute of Technology Ireland, Waterford, Ireland; 2 National Centre for Men’s Health, Institute of Technology Carlow, Carlow, Ireland

**Keywords:** men’s health, Gender-specific, Community, Implementation, Evaluation, Health promotion, Physical activity, men’s sheds

## Abstract

**Background:**

Men’s Sheds (“Sheds”) offer a unique opportunity to reach a captive audience of “hard-to-reach” men. However, attempts to engage Sheds in structured health promotion programmes must respect the ethos of Sheds as highly variable, autonomous, non-structured spaces. This paper captures the key methodologies used in “Sheds for Life’ (SFL), a men’s health initiative tailored to the Shed setting.

**Methods:**

A hybrid effectiveness-implementation study design is used to test effectiveness and implementation outcomes across multiple levels (participant, provider, organisational and systems levels). A dynamic, iterative and collaborative process seeks to address barriers and translation into the real world context. Using a community-based participatory research approach and guided by established implementation frameworks, Shed members (‘Shedders’) assume the role of key decision makers throughout the evaluation process to promote the systematic uptake of SFL across Shed settings. The protocols pertaining to the development, design and implementation of SFL and the evaluation of impact on participants’ health and wellbeing outcomes up to 12 months are outlined.

**Conclusions:**

There is a dynamic interplay between the intervention characteristics of SFL and the need to assess and understand the diverse contexts of Sheds and the wider implementation environment. A pragmatic and context-specific design is therefore favoured over a tightly controlled efficacy trial. Documenting the protocols used to evaluate and implement a complex multi-level co-developed intervention such as SFL helps to inform gender-specific, community-based men’s health promotion and translational research more broadly.

**Trial registration:**

This study has been retrospectively registered with the ‘International Standard Randomised Controlled Trial Number’ registry (ISRCTN79921361) as of the 5th of March 2021.

**Supplementary Information:**

The online version contains supplementary material available at 10.1186/s12889-021-10823-8.

## Background

### Men’s health – the need for gender specific approaches

Despite an increased emphasis on ‘the problem’ of men’s health in recent years, men remain disproportionately impacted by ill-health and premature mortality [1]. There have been calls for more gender-specific health promotion strategies that target lifestyle and health behaviour change, particularly to so called ‘hard-to-reach’ (HTR) groups of men (i.e. those who are unemployed, socially disadvantaged, isolated and have low educational attainment [2]. Paradoxically, these same groups are frequently the least likely to engage with health promoting initiatives [3, 4]. Early research into men’s health highlighted men’s avoidance of health promotion and health services as a consequence of aligning to more traditional traits of masculinity such as stoicism, self-reliance and competiveness [5]. More recently, the focus has shifted to positioning gender within a wider social determinants of health or intersectional context to better understand how gendered patterns of health behaviours are shaped by particular environmental, economic and socio-cultural contexts [6]. Such an approach acknowledges that men’s poor health outcomes reflect a multiplicity of factors that cut across all rungs of the social ladder and are exacerbated for vulnerable groups of socially disadvantaged or HTR men [7]. In fact, the biggest challenge for men’s health promotion is to better understand the complex biopsychosocial factors that influence men’s health in order to more effectively engage the most vulnerable men with health and wellbeing initiatives [8, 9]. Gender-specific approaches to health promotion need to account for the intersection between gender and other aspects of identity in designing tailored and targeted intervention that encourage men to engage [10, 11]. This approach also aligns more broadly with global health policies and priorities relating, for example, to the Sustainable Development Goals and reducing the burden on health systems [1, 12], and can also therefore be considered a strategic way of gaining momentum and support from policy makers and funders [10]. However, global gender equity policy still often fails to acknowledge men or else to position men and masculinities in a negative way, thereby creating challenges in translating knowledge into practice [13]. Within the Irish context there have been progressive movements to advancing men’s health equality. This is evident in a rich landscape of men’s health research and practice work that has emerged within Ireland in recent years [14], underpinned by a national men’s health policy [15, 16] and the roll-out of a national men’s health training programme [17, 18]. These serve as an important backdrop for men’s health promotion.

### Community-based Men’s health promotion in Men’s sheds

The non-clinical nature of the community setting is recognised as a key enabler of men’s engagement in health promotion programmes [4]. This setting allows a bottom-up, strengths-based, multi-sectoral approach that can effectively tackle the influence of more restrictive gender norms on men’s health behaviours, as well as providing men with a safe and familiar environment [19]**.** A range of additional gender-specific strategies have shown significant promise in engaging men, including; seeing men as partners, delivering key messages through informal approaches, identifying and utilising a ‘hook’ to engage men at buy-in stage, promoting positive social interaction and support, connecting more traditional masculine ideals (autonomy, control, resilience) with being healthy, using testimonials and peer support to encourage other men to take ownership of their health, actively seeking to promote camaraderie and team spirit, and drawing on language and styles that are relatable, [6, 11, 20]. These strategies are reflected in recent community based men’s health programmes such as; Men on the Move [21], the HATRICK programme [22], Famers have Hearts [23] and Football Fans in Training [24]. Utilising community settings for health promotion interventions while applying gender-specific strategies to engage more vulnerable male population groups, offer much potential in terms of easing the current burden on health systems [25]. These programmes provide a useful roadmap in designing and implementing health promotion in the Men’s Shed setting.

Sheds are a community-based, grassroots movement which originated in Australia and have since grown exponentially in Ireland. Sheds have long been recognised not just as a suitable setting in which to actively promote and engage men in health but also as being imbued with inherent and organic health promoting qualities [26]. Sheds are autonomous grass roots spaces which are non-structured and informal, varying in size and resources. Sheds offer a safe and familiar environment for Shed members (‘Shedders’) and foster a sense of social support, belonging and camaraderie, through developing new skills, shared projects, activities, goals and decision making [27]. All of these factors have been linked with enhancing the health and wellbeing of the men who attend with social support, in particular, being frequently reported as a key enabler of men’s help-seeking [28]. The enhanced sense of belongingness that is attributed to the non-conventional setting of Sheds increases their appeal to typically HTR men [29–31]. This is also the case in more recent research which suggests that the Sheds have a protective effect against loneliness [32]. The inherent health promoting qualities of Sheds therefore present a strong foundation upon which to build structured health promotion programmes. Moreover, drawing from a rich source of past interventions that utilised strengths-based and gender-specific approaches, the Men’s Sheds setting is well-positioned to deliver tailored, targeted health promotion initiatives to what has been traditionally regarded as an inaccessible of HTR cohort of men [33]. Nevertheless, it is also critically important that such endeavours enrich rather than erode the ethos of the Shed environment, which means that programmes need to be pragmatically evaluated with Shedders at the centre of decision making [33, 34]. Conventional wisdom dictates that health interventions need to be delivered systematically, to be context free, with strict inclusion criteria. However, Sheds are not just highly variable, autonomous, non-structured spaces; these are the very characteristics that define the essence of Sheds and which need to be respected in order to uphold the integrity and ethos of the Sheds. The challenge therefore is to develop a pragmatic delivery design that can operate within an organic, non-structured space, where contextual factors vary, where attendance can be sporadic, and where there is no compulsion on members to undertake any activity.

### Implementation science and the need for pragmatic evaluation

Preserving the integrity of the Shed environment and upholding the autonomy and respect of its members are key priorities that underpin any attempts to strategically evaluate health promotion programmes in Sheds. Findings from such evaluations are important in order to address the underrepresentation of men in health promotion programmes and to increase the availability of research that can act as a blueprint for practitioners and policy makers. It is important to capitalise on strengths-based and gender specific approaches by carrying out robust formal evaluations of these programmes [25, 26]. Furthermore, there is a lack of practical guidance on how to effectively plan, implement and scale up health interventions more broadly. Strategic and pragmatic evaluation endeavours encourage systematic uptake of effective interventions in real world settings, such as the Sheds, through limiting translation issues that can typically occur and prevent wider implementation of efficacious trials [35]. The challenges of implementing and sustaining health interventions in real world settings often emerge after tightly controlled efficacy trials are complete and conditions to disseminate and scale-up the interventions become much more variable [36]. A criticism of public health and health promotion research to date, is that barriers and facilitators to implementation in practice, such as the delivery capacity of partners and organisations, are often only addressed once the intervention is ready for wider implementation [37]. The result, often, is a failure to adopt and apply efficacious interventions to real-world settings. There have been calls for research to overcome this failure to translate evidence to practice by shifting the focus from tightly controlled interventions to evaluating those capable of implementation and scale-up from the outset [38]. The use of implementation science in the evaluation of health programmes can be valuable in identifying barriers to, and facilitators of effective implementation. By employing an iterative and collaborative process that engages with all key stakeholders across the implementation environment, it is more feasible to transcend barriers and translation issues in a pragmatic and dynamic way [38].

Whilst it is imperative to capture the ‘active ingredients’ of implementation and how they relate to each other, this can be challenging with more complex interventions [39]. It is important to remember that complexity is not just a property of the intervention but of the context or system into which it is placed, which includes multiple and dynamic interacting parts that generate nonlinear relationships [40]. Therefore, the potential effectiveness of health interventions is often reduced or poorly adopted because of multiple contextual factors that impact upon their implementation in real-life settings, such as the Sheds. In other words, knowing a health intervention is effective is not enough; there also needs to be a focus on understanding why and how it is effective to ensure that the model can be translated across implementation settings [41]. Hybrid-typology evaluation designs can therefore be a useful guide towards the dual testing of both clinical and implementation effectiveness particularly for community-based and real-world projects that can benefit from more rapid translational gains, more effective implementation strategies, and more useful information for decision makers [42]. This is particularly true of the Sheds setting where there exists a unique, naturally occurring opportunity to access a cohort of HTR men but where effective implementation strategies are critical within the variable, capricious, unstructured Shed environment. This paper addresses an important gap in the literature by applying an implementation lens to the evaluation of a community-based men’s health promotion programme using gender-specific approaches. The paper details the methodology used in the design, implementation and evaluation of the SFL programme. Specifically, it tracks the process of engaging men and delivery partners in SFL and sustaining their engagement over time, and it details the methods used to evaluate the impact of participation in SFL on various aspects of health up to 12 months.

## Methods

### Development of the “Sheds for Life” intervention in Men’s sheds

The concept of SFL was first developed in 2016 in response to a commitment from the representative body for Sheds (Irish Men’s Sheds Association; IMSA) to prioritise health initiatives for its membership. Prior to the implementation of a structured SFL programme, the IMSA embarked on scoping work at various Shed ‘Cluster meetings’ (regional information-sharing meetings with multiple Shed representatives). The purpose of this was to engage with Shedders on their health needs and their preferences for types of health promotion interventions in Sheds. This process confirmed that there was an appetite from Shedders for more structured health promotion that built on the inherent health promoting qualities of the Shed. The IMSA developed partnerships over time through on-going collaboration with various service provider organisations who were actively seeking to reach HTR groups of men in their health promoting endeavours and who had the capacity to deliver health and wellbeing components in the Sheds setting. This resulted in the piloting of a range of discrete health promotion initiatives in Sheds and to the emergence of SFL as a potential future health promotion programme for Shedders. In order to ensure that the goals of the IMSA and partner organisations aligned with Shedders’ needs, a research study was conducted at this time with key stakeholders (Shedders, IMSA, partner organisations) to explore their experiences of the SFL pilot programmes, and to reach consensus on an acceptable and respectful approach to deliver SFL in the Sheds [33]. The research found that respecting the Shed environment and its inherent health promoting values was critical to the acceptability of SFL. Involving Shedders in the decision making process of SFL, respecting the autonomy of the Sheds and tailoring SFL to the variable and individual settings of the Sheds were also highlighted as key priorities for Shedders. A fundamental requirement was to define a clear strategy and “rules of engagement” for implementing SFL and that those delivering elements of SFL understood and valued the ethos of the Sheds and its members [33]. Informed by this research, the IMSA developed a strategy document (“Guidance for Effective Engagement with Men’s Sheds”) to support health promoting organisations and professionals to respond and engage effectively with Shedders through SFL [43]. The document included a training workshop to support implementation of the guidelines during SFL delivery. In June 2018 the Irish Research Council awarded an Employment-Based postgraduate scholarship to support the formal evaluation of SFL. Over time, SFL evolved into a partnership network comprising the IMSA, [44] academics, an advisory group (consisting of men’s health promotion specialists and 12 allied service provider partner organisations), along with representation from Shedders.

### SFL Programme design

The findings of the SFL scoping study [33] guided the decision to structure SFL into a 10-week programme, which sought to deliver targeted and tailored wellbeing and life skill components to the Sheds. Four core components were identified aligning with the key pillars of the Healthy Ireland Framework and Healthy Ireland Men, including healthy eating, physical activity and mental health [16, 45]. Several optional components to accompany the core components were also developed to which Sheds could self-select, aligning with the needs of Shedders and the skillset of provider organisations (See Table 1 for an outline of SFL structure). This format was viewed by programme providers as being long enough in duration to encourage positive and sustained behaviour change, whilst from Shedders’ perspective, it also respected the fluid nature of Sheds in which a longer programme might conflict with Shed routine. Moreover this structure was pragmatic enough to consider whether SFL was feasible in the real-world, capricious Shed environment while prioritising future sustainability within existing funding structures. This structure and format were also informed by what worked in other programmes in Ireland with similar cohorts of men within community settings [21, 46]. Notwithstanding an agreed overall programme structure, careful attention was paid to how this worked in practice through a process of engagement with key stakeholders via formal and informal meetings, phone calls and emails which were ongoing through the pre-implementation and implementation phases of SFL. From January 2018 to January 2021, formal quarterly review meetings occurred with key stakeholders, at least twice weekly meetings took place between the health and wellbeing team responsible for co-ordinating SFL and the principal researcher, approximately 40 meetings occurred with individual provider organisations, and monthly report meetings took place with funding bodies, alongside quarterly financial reports.

**Table 1:** Structure of SFL phase 1 including workshops in development for phase 2 delivery

Core Components of SFL			
Programme Component	Description	Duration	Lead Provider
**Health check**	Health check by a registered nurse in a mobile health unit at the Shed measuring; Blood pressure, HR, cholesterol, carbon monoxide, weight, waist and body mass index	30 min at baseline	The Irish Heart Foundation
**Healthy Food Made Easy**	Basic nutrition & cookery course led by a trained facilitator	2.5 h workshops for 6 weeks	The Health Service Executive (HSE)
**Mental Health & Wellbeing in the Community**	Mental health and promoting positive wellbeing led by community development officer	4 h workshop (Available in 2 × 2 h session format)	Mental Health Ireland
**Sheds choose one of the two following physical activity programmes**:			
**Exercise for Shedders**	Indoor? Exercise class to maintain posture, strength, flexibility, balance & general physical capabilities led by qualified physical trainer	1 h exercise class for 10 weeks	Siel Bleu Ireland
**OR**			
**Sheds ag Siúl**^a^	Guided walking programme led by local sports partnership officer	1.5 h every second week across the 10 week programme	Get Ireland Walking
**Optional components of SFL**^b^			
**Diabetes: Living Well, Being Well Workshop**	Workshop on diabetes awareness and management led by qualified diabetes specialist	1.5 h single workshop	Diabetes Ireland
**‘Hands for Life’ CPR Training**	Workshop on performing CPR in the community led by CPR trainer	1 h single workshop	Irish Heart Foundation
**Oral Health**	Workshop on oral health awareness and maintenance led by dental nurse	1 h single workshop	Dental Health Foundation Ireland
**Cancer Awareness**	Workshop on cancer awareness & reducing the risk of male-related cancer led by cancer prevention officer	1 h single workshop	Marie Keating Foundation and Irish Cancer Society
**safeTALK**	Workshop on how to help prevent suicide by recognising signs, engaging someone and connecting them to an intervention resource for further support led by suicide prevention trainer	4 h single workshop	National Office for Suicide prevention Ireland
**Getting online computer training**	Beginners course on getting online & using devices led by trained community mentor	5 × 2 h sessions	Age Action Ireland
**Optional workshops in development for phase two delivery**^c^			
**Dementia Awareness**	Workshop to promote awareness of dementia signs, symptoms & risk factors & communication tips to support Shedders with early stage dementia in the Shed led by dementia advisor	1 h single workshop	Dementia Understand Together & The Alzheimer society of Ireland
**Bereavement and Loss**	Workshop to explore different forms of bereavement and loss as well as coping strategies for men led by bereavement specialist	TBC	TBC
**Sexual Health**	Workshop to explore male sexual health and relationships led by sexual health promoter	TBC	TBC

^a^Sheds ag Siúl: Walking component (‘ag Siúl’ Gaelic term for ‘walking’)

^b^Sheds select 2–3 optional components tailored to their Shed preference in addition to core components

^c^Shedders expressed a need during phase one implementation for new topics to be added to SFL & workshops that encompass these are currently in development for phase two

Although currently structured as a 10-week intervention with both core and optional components, SFL was designed as a flexible, dynamic programme, subject to ongoing adaptation to meet evolving needs. This meant that the SFL implementation strategy also needed to be flexible to accommodate new provider organisations over time in response to new or evolving requirements and preferences from Shedders. Thus, the structure and partnership network of SFL inevitably evolves and grows over time. Whilst this presents certain challenges, it can also be seen as a strength of the programme, not least in terms of its potential to remain fresh and contemporary. It is heavily invested in a partnership network that recognises the value of SFL and respects the ethos of Sheds. Also, SFL adopts a sustainable delivery model in that it is delivered under real-world conditions, where service provider organisations undertake SFL delivery as part of their routine work plans - as opposed to short-term (and often unsustainable) grant funding. That said, finite resources both in terms of a limited implementation workforce and competing priorities among provider organisations, demand that a prudent approach is taken to matching Sheds’ needs with programme offerings. The collaborative foundation of SFL where all key stakeholders are involved in decision-making is therefore an important consideration which can inform implementation outcomes and identify evolving implementation barriers and facilitators for early prioritisation.

### Engagement of HTR men using gender-specific implementation strategies

Health promotion initiatives that fail to incorporate gender perspectives into their implementation plans are usually less effective and, at worst, can perpetuate gender stereotypes that are not conducive to positive wellbeing [1]. The underpinning vision of SFL is to normalise conversations about health and wellbeing in Sheds and encourage help seeking, a vision that potentially conflicts with traditional norms of masculinity that are often regarded as being characteristic of more HTR groups [1]. Central to this approach is the positioning of Shedders as key stakeholders alongside provider organisations, researchers and the IMSA. This acknowledges Shedders as active participants in the overall process - from programme design to implementation to evaluation and indeed to informing strategies more broadly to engage HTR men in health. This also means investing in relationships, establishing credibility and tailoring new programmes around the needs of individual Sheds [33]. The implementation of the 10-week SFL format and application of implementation frameworks (see implementation research design) to guide the engagement process, also facilitates acceptability and optimises recruitment, participation and engagement in SFL.

The design and delivery of SFL draws heavily on established gender-specific approaches as outlined in section one of the introduction. These strategies are layered upon the male-specific, safe, familiar environment and sense of social support that is organic to Sheds. Among the key gender-specific strategies that are adopted for SFL are to (i) offer the programme free of charge, thereby removing cost barriers; (ii) provide a comprehensive health check as a “hook” to engage men; (iii) use non-typical health related components such as digital literacy and CPR as additional hooks to engage those less reluctant to sign up to a more conventional health programme; (iv) offer each Shed the choice (via an expression of interest form) to self-select into the programme based on Shed consensus, facilitating a sense of ownership, autonomy and control; (v) offer each Shed a selection of choice-based components, facilitating individual Shed preferences and further enhancing a sense of control and autonomy; (vi) use an informal and interactive delivery style to maximise engagement and enjoyment of the programme; (vii) foster an environment of openness and peer-support between participants; (viii) create a non-competitive and relaxed environment where participants engaged at their own pace; and (ix) visit each individual Shed in advance of the programme commencing, to brief Shedders on the programme, to build a sense of rapport and trust, and to assess the Shed environment’s suitability to participate in the programme (including adaptations needed to facilitate this). Sheds for Life is described to prospective participants as a programme “for Shedders by Shedders”. Prospective participants are encouraged to see themselves as pioneers, actively shaping the programme through their participation and paving the way for future delivery and scale-up of the programme. Reinforcing Shedders’ sense of ownership of the programme is designed to build safety and trust, and to reassure participants that SFL is not being implemented to undermine the routine environment and ethos of the Sheds, a critical factor in gaining acceptability at Shed level.

### Ethics, consent and data management

The study received ethical approval from Waterford Institute of Technology Research Ethics Committee (REF: WIT2018REC0010). This study has also been registered with the ‘International Standard Randomised Controlled Trial Number’ registry (ISRCTN79921361). During Shed visits, all participants have the details of the research clearly explained to them through verbal and written instruction and informed written consent is obtained by a member of the research team prior to participation in the research. Confidentiality of participants is ensured through the study’s compliance with Waterford Institute of Technology’s protection policy. Namely, all personally identifiable materials, such as consent forms, will be stored securely within the Institute. These will be stored separately from the transcribed research data and questionnaires, and only accessible by named researchers. All data sets will be kept on a password protected computer. Personal identifiable data will be retained for 5 y and then appropriately destroyed. Research data will be fully transcribed and anonymised, all details on identity, will be removed and replaced with deidentified information or pseudonyms. All enrolled participants will be allocated a unique study ID and the information linking their ID to their personal information will be held securely at Waterford Institute of Technology. All intervention content will be run under the guidance and training of IMSA by qualified external partners. Therefore, the risk to persons is not directly linked to this research. However, all SFL partners are adequately insured and qualified to run elements of SFL and engage in a screening process with participants to assess their ability to partake in the intervention for safety purposes. Screening elements of SFL will be run by registered nurses from the Irish Heart Foundation. Other practitioners working directly with participants are trained in first aid and also will complete Guidance training for working effectively within the environment of the Sheds. Topics covered in discussions or during questionnaire administration leave a small but important risk of participants becoming distressed. In case of such an event the distress of the participant will be ascertained, and the participant offered a break from the interview or to suspend the interview as appropriate. If a participant becomes distressed the researcher will stay with them until their distress reduces, or if their distress persists, the researcher will signpost them to an appropriate community support service. The researcher will report any issues of concern to the project supervisors and the IMSA.

### Effectiveness- implementation evaluation design

The SFL study adopts an implementation science focus. This approach strives to incorporate a broader scope than traditional clinical effectiveness alone; to focus not only on individual or participant level but also on how service provider, organisation, and wider systems impact implementation [36]. Successful implementation should be considered in light of a variety of factors including the effectiveness of the intervention to be implemented alongside implementation outcomes [41]. For these reasons, a hybrid type-two effectiveness-implementation study design was chosen. This means dual testing of effect and implementation outcomes of SFL in order to pragmatically promote translation into the real world context from the outset while also providing more valid estimates of potential effectiveness in the implementation setting of the Sheds [42]. In order to assess implementation outcomes and address barriers and facilitators to effective implementation, a community-based participatory research approach was adopted to involve key stakeholders across implementation levels [38]. Mixed methods are used to assess both implementation and effectiveness outcomes, which are described in detail in the following sections (See Table 2). The following sections outline the research design. Part 1 details how effectiveness of SFL is evaluated and Part 2 describes how the SFL implementation is evaluated.

**Table 2:** SFL Effectiveness-Implementation Hybrid design

Evaluation	Research Question	Research Methods & Data collection approaches	Tools & Frameworks	Targeted Outcome
**Implementation**	What are the facilitators and barriers that impact implementation and sustainability of SFL across the individual, provider, organisation and wider systems level?	Qualitative Participatory Research Focus groups, Interviews (Hybrid approach of thematic deductive and inductive analysis) Ethnography Stakeholder meetings	PRACTIS guide (PRACTical planning for Implementation and Scale-up) CFIR (Consolidated Framework for implementation research) Semi-structured topic guides	Acceptability Adoption Appropriateness Feasibility Fidelity Implementation Cost Penetration Sustainability
	What is the process by which the SFL model is developed and implemented in order to effect maximum penetration, adoption and acceptability among key stakeholders?	Qualitative Participatory Research Focus groups, Interviews Ethnography Stakeholder meetings Quantitative Recording attendance (providers) & Self-reported attendance at follow-up (participants)	PRACTIS guide (PRACTical planning for Implementation and Scale-up) CFIR (Consolidated Framework for implementation research) Attendance and membership records Semi-structured topic guides	Penetration Adoption Acceptability
	How does the Partnership and Capacity building focus of SFL contribute to the implementation and scale-up of the programme?	Qualitative Participatory Research Interviews Stakeholder meetings Capacity Building Workshops	PRACTIS guide (PRACTical planning for Implementation and Scale-up) CFIR (Consolidated Framework for implementation research) Semi-structured topic guides	Acceptability Adoption Appropriateness Feasibility Sustainability
	Is the SFL implementation approach cost-effective?	Quantitative Cost Gathering Assessment of cost using Quality Adjusted Life Years	The SF6D	Implementation Cost Feasibility Sustainability
**Effectiveness- Implementation**	Does participation in Sheds for Life improve health knowledge attitudes, outcomes and behaviours among participants?	Pragmatic controlled Trial Quantitative Questionnaires administered at baseline, 3, 6 & 12 months Qualitative Focus groups, ethnography, key informant interviews	Core outcome tools Self-reported Health Rating Seeking health information rating 7-item Short Warwick- Edinburgh Mental Wellbeing Scale (SWEMWBS) 5- point Likert Scales assessing; comfort having a conversation about mental health, understanding mental health and identifying practical supports 3-Item UCLA Loneliness Scale. Rated on a 3- point scale. Higher scores equal increased loneliness ONS 11-point Scales 0–10 Life satisfaction and life worth 8 point scales 0–7 physical activity and walking measure The 9-item self-efficacy for exercise scale (SEE) Close support, belonging, trust Alcohol, smoking & fruit & vegetable consumption Cooking frequency, cooking style and 12 measure scale measuring confidence constructs in relation to cooking Supplementary outcomes: Questions measuring changes in confidence and knowledge in relation to supplementary components developed in collaboration with provider organisations Qualitative tools Semi-structured topic guides	Quantitative Core outcomes Subjective Wellbeing Help-seeking Physical Activity Mental Wellbeing Diet & Cooking skills Social Capital Self-efficacy Quantitative Supplementary outcomes Diabetes Awareness, SafeTALK suicide prevention, Digital Literacy, Oral Health, Cancer awareness, CPR Qualitative outcomes Changes in attitudes and behaviours Acceptability Adoption Appropriateness

### Part 1: evaluating the effectiveness of SFL-research design

#### Overview

Phase one of SFL encompassed the first delivery of the programme in Sheds. Following assessment of the implementation environment, namely the capacity and resource constraints of provider organisations to deliver SFL along with the nuances, ethos and autonomy of the inner (Sheds) setting, the SFL 10-week intervention was implemented on a phased basis across two cohorts comprising two counties in each cohort with a view to delivering Phase 2 as a single cohort across a further four new counties (i.e. Phase 1 (4 counties, two cohorts); Phase 2 (4 counties, one cohort); see participants and sampling). A mixed methods approach was applied to assess the impact of SFL Phase 1 testing on the biopsychosocial health of participants up to 12 months. This consisted of focus groups, interviews and questionnaires assessing health outcomes.

#### Participants and sampling

Respecting the autonomous and informal environment of the Sheds is an important factor in delivering health promotion through Sheds [27, 33]. Therefore, Sheds are recruited to participate in the SFL programme and evaluation via purposive sampling using an expression of interest process with the objective to deliver SFL in a diverse range of Shed settings (small/large, urban/rural). All adult males in the Sheds setting were eligible to participate in the study providing they had good proficiency of the English language and could give informed consent. A sample size estimation was undertaken using G*Power 3.1.9.2 software using physical activity (PA) as the key outcome measure, whereby it was calculated that 106 participants would be required for a trial in which participants were individually randomised (the decision to use PA as the primary outcome measure was determined through consultation with Shedders who requested that PA be a key focus of SFL during the initial pilot phase). However a clustered design in which SFL was delivered to small clusters of men within Sheds was more preferable to honour the Shed ethos whilst also ensuring a wide geographical spread. For this reason a design with circa 20 men in each cluster was estimated. A previous study with middle-aged men suggested that this design effect is ~ 2.4, thus increasing the sample size required to 255 [47]. Allowing for a 20% dropout based on a sample size estimation, the final total required was 306 or 15 clusters. In the event of low participation within clusters, it was decided that SFL would be targeted at clusters with similar representation. In Phase 1, 421 Shedders participated across 22 clusters and these were divided into two cohorts. Whilst delivery occurred in the first cohort (*n* = 12 clusters; *n* = 212 Shedders) a wait list control cohort served as a comparator (*n* = 3 clusters; *n* = 89 Shedders) and these were a subset of the second cohort (*n* = 9 clusters, *n* = 209 Shedders). Fourteen clusters were in urban areas and 8 were in rural areas across counties; Kildare (in Ireland’s mid-east region with a population of ca. 222, 504), Waterford (in Ireland’s south-east region with a population of ca. 116,176), Limerick (in Ireland’s south-east region with a population of ca. 194,899) and Louth (in Ireland’s mid-east region with a population of 128, 884) [48]. Participants were recruited for Phase 1 across Waterford and Kildare in March to May 2019 and Limerick and Louth in September to December 2019. Participants for Phase 2 will be recruited from September to December 2021 (recruitment was postponed until this date due to COVID-19 restrictions).

Purposive sampling was also used to conduct formal focus groups (*n* = 8) with participating Sheds in Phase 1. This qualitative study seeks to gather a diverse representation of Shedders’ experiences of SFL to compliment quantitative findings including changes in knowledge, attitudes and behaviours. Informal short interviews (*n* = 16) were also conducted ad-hoc during Shed visits in Phase 1 to further inform Shedders’ experiences of SFL.

#### Evaluating the effectiveness of SFL- data collection

Questionnaires are administered to participants by a research team member trained in data collection procedures to ensure standardised measurement and questionnaire administration. Questionnaires are administered one-to-one in the Sheds setting to account for potential literacy issues, prevent respondent burn-out, limit missing data and build rapport and trust between the researchers and Shedders. To also minimise missing data, participants will be contacted by the IMSA in the days before the research team visit the Sheds to perform data collection. Due to the informal nature of the Sheds, absence of data for a participant does will not necessarily indicate dropout from SFL. During 6 and 12 month follow-up in Phase 1, Cohort 2 were experiencing COVID-19 restrictions and therefore questionnaires were administered via phone in order to promote participant retention and complete follow-up. The questionnaire was designed via a consultation process with stakeholders involved in the design and delivery of SFL with a view to optimising acceptability for SFL participants and also SFL providers who were interested in evaluating their individual components of SFL. Participant demographics are recorded at baseline and include date of birth, living arrangements, educational attainment, employment status relationship and ethnicity. Participants are also asked how long they had been a Shed member and how often they attend the Shed. Core health and wellbeing outcomes measured at all-time points up to 12 months consist of; subjective wellbeing, help-seeking, physical activity, mental wellbeing, diet and cooking skills, social capital and self-efficacy. Participants are also asked how often they seek information about their health.

Self-rated health is measured using a single question Likert scale with high reliability among older men [49]. The single-item PA measure is used to record PA levels [50]. The Self-Efficacy for Exercise Scale (SEE) is used to measure physical activity self-efficacy [51]. Life worth and satisfaction are recorded using the Office of National Statistics subjective wellbeing 11-point scales [52]. Mental wellbeing is measured using the Short Warwick-Edinburgh Mental Wellbeing Scale (SWEMWBS) with raw to metric score conversion where a change of 2+ is considered relevant [53]. Loneliness is measured at all-time points using the UCLA 3-item scale measuring three dimensions of loneliness; relational connectedness, social connectedness and self-perceived isolation, with participants also asked at baseline to retrospectively rate their loneliness prior to joining the shed [54]. Social Capital is measured based on relevant recommendations from WhatWorksWellbeing [55], capturing trust, belonging and close support. Interpersonal trust is measured using the Office of National Statistics 11-point scale [52]. Lifestyle behaviours are also recorded - smoking (number smoked per day) and alcohol consumption (days drinking and units consumed per drinking session). Assessments of cooking and healthy eating behaviours are developed in conjunction with the partner organisation delivering the Healthy Food Made Easy component of SFL. Participants are asked about their levels of daily fruit and vegetable consumption, cooking style, cooking frequency and willingness to cook. Confidence constructs around cooking and healthy eating are measured via a 12 item Likert scale ranging from “not at all confident” to “very confident”. The questions were adapted from a protocol for community-based cooking interventions which were developed at a lower literacy level with varying levels of literacy among participants in mind [56]. The constructs used to assess cooking and healthy eating were previously validated [57] (See Table 2 for effectiveness outcome measures including optional components).

Semi-structured topic guides were developed for focus groups and short interviews. These were designed using a hybrid deductive-inductive approach applying implementation frameworks to assess implementation outcomes but also to allow room for exploring attitudes towards SFL, changes in knowledge and behaviours. A constant comparison process is being used to refine and adapt topic-guides to reflect new themes to be explored as SFL evolves across implementation settings.

#### Evaluating the effectiveness of SFL- data analysis

Questionnaire data is analysed using Statistical Packages for the Social Sciences (SPSS V 24). Descriptive statistics for each variable are calculated to inform participant characteristics. Intervention effect on health and wellbeing outcomes are determined by comparing the change scores from baseline to 3, 6 and 12 months, comparing data using inferential tests to identify significant differences set at *p* = 0.05. Analysis of subgroups based on criteria such as; Shed size location and timing of the intervention, will also be performed to identify significant differences in intervention effect between groups. Outcome data obtained from all participants are included in the data analysis, regardless of adherence to SFL. The intervention effects will be assessed at 3, 6 and 12 months based on those who completed follow-up at these time points. Assuming a worst case scenario for absentees i.e. that absentees failed to achieve a significant improvement in core health outcomes (physical activity, diet, mental wellbeing, subjective wellbeing, social capital and help-seeking), these worst- case scenario analyses will reflect the intention to treat principle. The numbers who achieved significant improvement at 3, 6 and 12 months will be presented as a percentage of those who were tested at these follow-up points. For the initial intervention effect worst-case scenario, the numbers who achieved significant improvements at 3 months will be presented as a percentage of those who were tested at baseline. The worst-case scenario for maintenance of this initial intervention effect will present the numbers who achieved significant improvements at 6 and 12 months as a percentage of those who were tested at the 3 month follow-up. Observed success rates will be compared between the intervention and comparison group in waiting using Chi-Square analysis.

A hybrid analytic approach of inductive and deductive analysis is applied to the participant transcripts. This means that whilst implementation frameworks are applied to inform implementation outcomes, the analysis process will remain open to findings that may emerge outside of those pre-set domains to allow assessment of intervention effect. In these circumstances, inter-rater reliability is used to cross-check coding strategies and interpretations are negotiated to agree on a ‘master’ code list.

### Part 2: evaluating the implementation of SFL- research design

#### Overview

The implementation and sustainment of an effective, evidence-based programme in the real-world setting is complex and therefore multiple frameworks are increasingly being used in studies to address multiple facets of implementation [58, 59]. Sheds for Life operates within a complex system of shifting elements such as the diverse and variable contexts of the Sheds and the wider implementation environment, including the competing priorities of provider organisations and systems level funding and polices. As a result, there is a need to continually engage current and emerging stakeholders as well as inform key adaptations and processes that are necessary to implement SFL in multiple locations while executing appropriate implementation strategies to embed SFL in the routine environment of the Shed. Recognising the context in which SFL is implemented as a constellation of active intervening variables rather than simply a backdrop for implementation is therefore important to better identify and address implementation challenges [60, 61]. Indeed, these dimensions continually evolve over time and require on-going monitoring. For this reason, a combination of implementation and evaluation frameworks is used to guide the implementation testing and evaluation of SFL. These frameworks consist of a determinant framework to specify constructs that may influence the SFL process and predict implementation outcomes, a process framework to specify steps to execute for implementation phases and an evaluation framework to specify multiple levels of outcomes to assess [59].

The determinant framework used is The Consolidated Framework for Implementation Research (CFIR) [60]. This framework is used to characterise and understand constructs across five domains which interact in complex ways to influence implementation outcomes. These include; i) the characteristics of the SFL intervention (e.g. how complex the intervention is), ii) the outer setting (e.g. external policies that influence the SFL intervention), iii) the inner setting (e.g. the readiness for SFL implementation), iv) the characteristics of individuals (e.g. individual self-efficacy), and v) the intervention process (e.g. engaging individuals to champion SFL). The CFIR was used as a practical guide to systematically assess potential barriers and facilitators in preparation for implementing SFL. It was also used to develop topic guides for stakeholders at each level to characterise the implementation setting during SFL implementation as well as to guide the observation of SFL.

The process framework applied to SFL implementation is the (PRACTIS) - PRACTical planning for Implementation and Scale-up guide [38]. The PRACTIS is used in an iterative process to practically guide the implementation process and evaluation in collaboration with key stakeholders. In this study, it is used to promote successful implementation and scale-up of SFL. Sheds for Life implementation is guided by four key steps, namely; characterising the parameters of the implementation setting, identifying and engaging key stakeholders, identifying implementation barriers and facilitators, and addressing potential barriers to implementation across individual, provider, organisational and systems levels. The implementation setting is characterised by following a checklist criteria of 5 P’s i.e. i) People; the individuals involved for effective implementation of SFL, ii) Place; what settings and organisations with be involved in SFL iii), Process; how the implementation process of SFL will occur iv), Provisions; what resources may be necessary to achieve this process, and v) Principles; what are the underlying principles of SFL and the implementation process that will be scaled-up. These were explored in collaboration with key stakeholders as per PRACTIS [38]. Additional File 1 demonstrates SFL operationalisation of the PRACIS guide (See Additional File 1).

Finally, the evaluation framework applied to SFL is the taxonomy for implementation outcomes [41]. This framework measures outcomes pertaining to implementation i.e. acceptability, adoption, appropriateness, feasibility, fidelity, implementations costs, penetration and sustainability. These are assessed in the SFL evaluation using mixed methods to measure implementation effect. Implementation testing consists of ongoing engagement with service provider organisations through quarterly stakeholder meetings, observation and field notes, interviews and focus groups as well quantitative measures to assess cost outcomes (See Table 2).

#### Evaluating the implementation of SFL -data collection

In order to explain or understand implementation outcomes, the perspectives and experiences of a broad representation of stakeholders at the participant, provider, organisation and wider systems level are sought. Purposive sampling is used to identify key informants for interview to inform implementation outcomes across the multi-level implementation environment. Mixed methods are used to inform implementation outcomes. The PRACTIS guide is used as part of an iterative process to characterise parameters of the implementation setting, engage key stakeholders, identity implementation barriers and facilitators and address potential barriers to implementation within the evolving implementation climate [38]. Ongoing consultation with stakeholders is deemed appropriate to the implementation approach as contextual shifts can be unpredictable and assessment of the broader implementation environment requires flexibility and iteration [62]. Alongside this, interviews (*n* = 19) at provider, organisational and systems level are also conducted using semi-structured interview schedules which are designed based on CFIR constructs and used to inform a taxonomy of implementation outcomes, with room for other themes to emerge [41, 58]. Focus groups and interviews previously outlined at participant level are also used to inform implementation outcomes. As a considerable amount of time is spent in the variable environments of different Sheds during data collection, observation and field notes are also used to discover and document the context in which implementation occurs. This process is guided by CFIR constructs with a view to also informing the effectiveness of implementation strategies.

The questionnaires administered to Shedders at baseline, 3, 6 and 12 months are also used to inform implementation outcomes; cost and penetration of SFL. Self-reported attendance records are collected at follow-up points via the questionnaire to capture attendance. Providers of the SFL components also capture attendance records at delivery and records of the numbers of Shedders who are eligible versus those who participate in SFL are gathered to further inform penetration. The short form 6-D (SF-6D) is assessed via the questionnaire, alongside the gathering of cost data for assessing cost effectiveness of SFL. It is a preference-based measure of health with a six-dimensional health status classification: physical functioning, role functioning, social functioning, pain and discomfort, mental health and vitality. It was derived from the SF-36. Participants select one of the levels (up to level 4 or level 6) in each dimension which best describes their current health status [63].

#### Evaluating the implementation of SFL -data analysis

Data pertaining to SFL participation (attendance records, self-reported attendance, numbers who participated versus numbers eligible) are triangulated to assess penetration. Cost-effectiveness is being determined by comparing the costs (direct and indirect) of SFL to its benefits which will be captured as the impact on quality-adjusted life-years (QALYs) derived from the short form-6D algorithm. Qualitative data are analysed using a framework-driven approach, applying the CFIR to inform implementation outcomes. Focus groups and interviews will be transcribed and, as per recommendations by the National Cancer Institute’s White Paper on qualitative research in implementation science, a hybrid approach of thematic deductive and inductive analysis will be used to inform implementation outcomes [44]. This means that whilst the CFIR domains will be applied to inform implementation outcomes, the analysis process will remain open to findings that may emerge outside of those pre-set domains. A constant comparison process previously outlined will again be applied.

### Limitations

While the non-randomised design of SFL may be seen as a limitation, the SFL research exists within a complex real-world environment with many evolving variables. For this reason, a pragmatic evaluation approach is necessary in which upholding Shed ethos means that participants cannot be randomised for assessment of intervention effect. However a strength of this approach is also in the process of identifying an appropriate implementation model that can effectively engage HTR men with targeted health promotion in the capricious Sheds environment. The very nature of this environment is what attracts HTR men and for this reason it is critically important that this informal and autonomous atmosphere is maintained when synchronising with more structured health promotion. There is also a subjective nature to the data that allows inherent bias through the self-report format. Yet, constructs of wellbeing and perceived health status are subjective in their own right and the evaluation captures insights from Shedders in the real-world context of a typically close-knit setting.

## Discussion

An important backdrop to SFL is the rich landscape within its outer setting of men’s health research and practice work that has emerged within Ireland in recent years [14, 64]. While SFL evolved mostly as a bottom-up initiative to address a particular need, it was also mandated by a top-down men’s health policy directive [16]. This wider context of men’s health work within Ireland was highly conducive to and compatible with the key principles and objectives of SFL. Crucially however, SFL was not foisted on Shedders! On the contrary, SFL emerged from an invested process of engagement, consultation, relationship building and pilot testing. These efforts seeded partnership networks that understood the processes and recognised the value in engaging men with health. This is an important consideration at a time when Sheds have been earmarked as settings that facilitate access to HTR men and where expectations placed on Sheds to expand into formal healthcare delivery may cause tensions within Sheds [34]. While the content and structure of SFL may evolve over time, this process of delivery and partnership are the crux of its sustainability. Sheds for Life operates within a systems level that does not yet offer any significant funding support but the partnership and capacity building processes of SFL remain the crucial elements in terms of its sustainability.

Sheds for Life challenges traditional gender norms about health by reframing men’s active engagement in their own health and encouraging male peer support in dealing with health issues as a socially acceptable and ‘manly’ choice [1]. Through this process of engagement, SFL reflects a gender-transformative approach, normalising health conversations within the culture and environment of Sheds – settings that have not traditionally prioritised health and wellbeing. This also challenges gender stereotypes of women as care-givers and custodians of men’s health, thereby contributing to gender equality. The efforts to shift health programmes with men from being gender-neutral to more gender-specific and gender-transformative, can improve population health for both women and men by enhancing equitable gender relations [65]. It is evident that the burden of ill health in men is caused by a multitude of complex biopsychosocial factors. In order to address gender inequality in health, movements towards the development of health promoting strategies and interventions that account for the diversity within and between genders are critical to advancing population health [66]. In this respect, reaching beyond the ‘worried well’ and engaging HTR groups of men remains a key priority. Effective men’s health programmes to date have highlighted that, in order to engage men, and particularly those who are considered HTR, health promotion endeavours must include men in their decision making and encourage a collaborative process involving all key stakeholders; researchers, practitioners, participants and policy makers [9, 34, 65].

The SFL evaluation investigates both the implementation and effectiveness of the intervention and identifies the key strategies to engage HTR men in health within the non-conventional settings of the Sheds. Research findings from complex interventions to promote health suggests that traditional research and practice methods fall short in meeting many of the challenge inherent in complex interventions. This means that science needs to reassess some essential beliefs and prejudices about research methods and conventional terminology which is overly focused on knowledge generation and can blind researchers to the very mechanisms they seek to understand within the practice context [40]. The SFL evaluation embeds implementation processes and outcomes from the start with active engagement from all key stakeholders – including Shedders. The move from tightly controlled trials towards pragmatic delivery in the real-world Sheds setting using a bottom-up, multi-sectoral approach is key to identifying implementation strategies within the continually shifting context that can promote systematic uptake of SFL. Scale-up within a complex environment can mean that programmes may rarely be translated to variable settings completely intact or standardised [40]. Rather it is the “core principles” that are essentially transferred and knowing this in advance can encourage the essence of an intervention to be distilled while applying more conscious processes to enhance its effects and embed it into the routine environment [40]. The flexible implementation strategy of SFL outlined in this paper highlights how the structure and partnership network of SFL will evolve over time. The sustainability of SFL in the variable Sheds settings will mean adaptations to suit the local contexts in which it operates. In its scale-up, the evaluation will aim to protect the essence of SFL by translating the core principles of the programme, viewing its fidelity as residing in the theory of the change process (i.e. the changes in the Sheds context which bring about, aid or sustain individual change) rather than in any particular component [40]. The SFL approach aims to effectively promote positive men’s health behaviours in what men consider a safe and familiar environment. It also aims to encourage intervention development and adaptation of SFL that ensures broad and sustained implementation. This approach is explicitly orientated towards delivering impact-focused research activity that forges strong links between research and practice. Findings will have a significant role to play in determining the effectiveness, sustainability, and potential scale-up of the SFL initiative and, more broadly, in terms of the wider translation of community-based programmes targeted, in particular, at HTR groups of men. This study provides many excellent opportunities for knowledge translation that can have a tangible impact on practice in the fields of health promotion, public health and men’s health.

### Dissemination

SFL is grounded in implementation science and therefore results of the study will be disseminated to key stakeholders on an ongoing basis in order to inform necessary adaptations. An interim analysis will be performed following Phase 1 implementation to assess the impact of SFL on the health and wellbeing outcomes of participants. These findings will be made available in an impact report document that will be accessible to participants, provider organisations, the IMSA and any other relevant groups. Some interim findings have been also been reported in published work relating to the impact of COVID-19 on Shedders [32]. Funders will play no role in the study conduct, analyses or data interpretation. There are no publication restrictions and findings of the research will be widely disseminated. Key outputs from SFL implementation will contribute to the dissemination plan. Data from Phase 1 testing will inform Phase 2 implementation. It is envisaged there will be numerous publications arising from this research study along with presentations at national and international conferences. The findings from SFL will be used to produce a final report for the IMSA targeted principally at policy makers and service providers. An accessible version of the report will be produced for Shedders and the general public to ensure knowledge exchange at all levels.

## Supplementary Information

**Additional file 1.**

## Data Availability

Not applicable.

## Abbreviations

SFL: Sheds for life
Shedders: Men’s Shed members
Sheds: Men’s Sheds
HTR: Hard-to-reach
IMSA: Irish Men’s Sheds Association
PRACTIS: PRACTical planning for implementation and scale-up
CFIR: Consolidated framework for implementation research
SWEMWBS: Short Warwick-Edinburgh Mental Wellbeing Scale
PA: Physical activity
SF-6D: Short Form 6D
