# Supplementary material for: Study protocol: evaluation of sheds for life (SFL): a community-based men’s health initiative designed “for shedders by shedders” in Irish Men’s sheds using a hybrid effectiveness-implementation design
Source: BMC Public Health. 2021 Apr 26;21:801. doi: 10.1186/s12889-021-10823-8 (PMC8072742; doi:10.1186/s12889-021-10823-8)
Supplement: Supplementary file 1 — Additional file 1. [file 12889_2021_10823_MOESM1_ESM.zip › Addiotnal File 6 All items WHO Trial Reg Data SetR0.docx]

All items from the World Health Organization Trial Registration Data Set

| **Data category** | **Information** |
| --- | --- |
| Primary registry and trial identifying number | International Standard Randomised Controlled Trial Number (ISRCTN79921361) |
| Date of registration in primary registry | 5^th^ March 2021 |
| Secondary identifying numbers | N/A |
| Source(s) of monetary or material support | Irish Research Council |
| Primary sponsor | Irish Research Council (Project ID EBPPG/2018/256). |
| Secondary sponsor(s) | N/A |
| Contact for public queries | Dr. Noel Richardson, National Centre for Men’s Health, Institute of Technology Carlow Email: noel.richardson@itcarlow.ie |
| Contact for scientific queries | Dr. Noel Richardson, National Centre for Men’s Health, Institute of Technology Carlow Email: noel.richardson@itcarlow.ie |
| Public title | Shedding light on men’s health: Evaluating the scalability of a community-based men’s health promotion programme through the application of implementation science |
| Scientific title | The effect of a 10-week gender-specific men’s health intervention on health and wellbeing outcomes of Irish Men’s Shed members |
| Countries of recruitment | Ireland |
| Health condition(s) or problem(s) studied | Promotion of physical activity, subjective wellbeing, mental-well-being and healthy diet in Men’s Shed members |
| Intervention(s) | 10 week men’s health initiative consisting of;  Four core pillars including: 1. An initial free health check [BMI, waist circumference, BP, cholesterol, glucose and carbon monoxide] 2. Structured 1 hour weekly exercise of either a) a facilitated group walking program or b) group exercise focusing on strength, balance and mobility 3. A facilitated four hour mental health workshop 4. A cooking and health eating course (2.5 hours weekly for six weeks)  The intervention consists of several other facilitated, optional workshops that Sheds can select including; CPR, suicide prevention, diabetes awareness, cancer awareness, digital literacy, bereavement, dementia awareness and oral health awareness. The core objectives of the intervention are standardised across delivery sites. |
| Key inclusion and exclusion criteria | Inclusion criteria: Adult males in the men's shed setting  Exclusion criteria: Non-proficiency in the English language |
| Study type | Interventional Multicenter longitudinal pragmatic controlled trial Primary purpose: prevention |
| Date of first enrolment | 04/03/2019 |
| Target sample size | 600 |
| Recruitment status | Recruiting Recruitment end date 06/09/2021 |
| Primary outcome(s) | Time frame: 12 months   1. General health history, help-seeking and perception 2. Self-rated health 3. Changes in physical activity 4. Physical activity self-efficacy 5. Subjective wellbeing 6. Mental wellbeing 7. Social Captial 8. Dietary habits 9. Alcohol and smoking |
| Key secondary outcomes | 1. Cost-efftectiveness 2. Assessment of implementation outcomes 3. Assessment of optional intervention components, tracking changes in confidence, knowledge and attitudes assessed at baseline, 3, 6 and 12 months |
