# Supplementary material for: Study protocol: evaluation of sheds for life (SFL): a community-based men’s health initiative designed “for shedders by shedders” in Irish Men’s sheds using a hybrid effectiveness-implementation design
Source: BMC Public Health. 2021 Apr 26;21:801. doi: 10.1186/s12889-021-10823-8 (PMC8072742; doi:10.1186/s12889-021-10823-8)
Supplement: Supplementary file 1 — Additional file 1. [file 12889_2021_10823_MOESM1_ESM.zip › Additional File 1 PRACTIS GuideR0.docx]

**Additional File 1: SFL operationalisation of the PRACTIS guide**

| Operationalisation of the PRACTIS guide to identify process of SFL implementation | |
| --- | --- |
| Intervention: Men’s Health and Wellbeing programme “Sheds for Life” | |
| *Target outcome* | *Engaging HTR men with health and wellbeing. Improve knowledge, attitudes and health and wellbeing outcomes (PA, Diet, Mental Wellbeing)* |
| *Population*  *Setting* | *Community-based men’s health programme in the men’s shed setting. Target group: Men’s Shed members* |
| Step 1: Characterize implementation setting parameters | **Place**: Targeted Intervention delivered directly in the men’s shed setting  **People** & **Process**: 10 week gender-specific, structured intervention targeting Men’s Shed members across four counties. Consists of core pillars of a health check, healthy eating, physical activity and mental health with several other optional components tailored to individual Sheds. Participating Sheds “self-select” into SFL via an expression of interest process and individual Shed members are actively recruited by research team and IMSA by visiting individual Sheds.  Delivered by allied provider organisations whose ethos align with the goals of the IMSA and can effectively respond to the needs of Men’s Shed members. Organisations who have participated in the Guidance for Effective Engagement with Men’s Sheds training and understand and respect the ethos and environment of the Sheds.  **Provisions**: Training workshop and manual for facilitators, expression of interest forms for participant recruitment, SFL handbook for participants, attendance records, and attendance certificates. Supplementary resources. Text-based reminders and programme calendars. Self-reported questionnaires administered by trained researchers.  **Principles**:  *Intervention* –  Engage HTR men with health, normalise conversations about health in the Shed environment. Enhance knowledge and awareness about health topics. Improve health outcomes; subjective wellbeing, diet, physical activity, mental health, social capital.  *Implementation* – Targeted intervention delivered within the shed setting. Uses existing infrastructure of Sheds where men naturally congregate and builds upon its inherent health promoting qualities such as the male-specific environment, sense of safety and social support. |
| Step 2:  Identify and engage key stakeholders | Stakeholders represented the participant, provider, organisational and community/systems levels. Created a stakeholder group (Organisations delivering elements of SFL, academics, the IMSA and funding bodies), to guide study design, evaluation and intervention protocol and adaptation. |
| Step 3:  Identify contextual barriers and facilitators | Org. and provider level barriers: i) capacity of IMSA staff and providers to implement  Systems level: ii) sustained funding body  User characteristics iii) variable Shed settings, engaging HTR men, beliefs about health and wellbeing |
| Step 4: Address/assess barriers | *Formative evaluation*: Scoping work in Sheds informed blueprint for acceptable delivery model of SFL. Formation of strategic partnerships to respond to needs of Shedders. Piloting of SFL elements in Sheds. Identification of suitable providers to implement SFL in participating counties. Capacity building focus to ensure providers understood delivery approach. Training manuals and workshops developed and implemented for providers. Stakeholder group provided feedback to guide development of intervention processes and materials. SFL structured into a ten-week intervention. Implementation and evaluation designed in collaborative process.  *Strategies to address barriers*:  Community based participatory research approach employed to work closely with provider level stakeholders in ongoing process to address capacity barriers and identify suitable facilitators for implementation on the ground.  Assessment of suitability of potential funding streams.  Design of implementation and gender- specific strategies to maximise participation in SFL. Including Shedders as both active participants in the research and programme. Assessment of individual Shed settings to inform adaptations required for effective implementation  *Process/outcome evaluation*:  Effectiveness-implementation hybrid approach to assess the impact and implementation outcomes of SFL to promote the systematic of the intervention across individual, provider, organisational and systems level.  *Economic evaluation:*  Cost-effectiveness analysis of SFL to demonstrate that the intervention impact and value for money of SFL to prospective funders. |
